# Patient Monitor Position and Operator Ability to Visualize a Desaturation Event During Intubation

**DOI:** 10.5811/westjem.52933

**Published:** 2026-06-16

**Authors:** Eric Boccio, Rachelle Perkins, James Bonz

**Affiliations:** * Mount Sinai Medical Center of Florida, Department of Emergency Medicine, Miami Beach, Florida; † Intermountain Medical Center, Department of Emergency Medicine, Murray, Utah; ‡ Yale New Haven Hospital, Department of Emergency Medicine, New Haven, Connecticut

## Abstract

**Introduction:**

During endotracheal intubation, equipment setup typically includes the patient monitor fixed at the head of the bed behind the back of the operator. Inability to directly visualize the patient monitor may result in delayed recognition of desaturation. Our primary aim in this study was to measure the association between patient monitor position and the time to recognition of a desaturation event during endotracheal intubation.

**Methods:**

We performed a randomized crossover trial of emergency medicine residents across two Accreditation Council for Graduate Medical Education-accredited programs. Subjects were asked to perform direct and video-assisted laryngoscopy once on a difficult airway trainer in a simulation. The sequence of laryngoscopy modality (direct vs video-assisted) and monitor position (head vs left vs right of bed) were randomized prior to each attempt. The simulated patient’s peripheral capillary oxygen saturation (SpO2) was programmed to begin at 100% and decrease at a rate of 1% per second 10 seconds after the start of the subject’s attempt. The primary outcome measure was time to operator recognition of hypoxia defined as the observed period during which the simulated SpO2 was < 90%. Secondary outcomes were operator failure to visualize a desaturation event. We rendered Kaplan-Meier curves illustrating the time to visualization of hypoxia and performed a Cox regression adjusting for laryngoscopy modality and total number of previous intubations performed. Using multivariable linear regression, we modeled the association between time to recognition of hypoxia in seconds and patient monitor position with similar adjustments. To assess differences in the number of observed failure events between study arms, we used chi-squared or Fisher exact tests. The threshold for statistical significance was a two-sided P < 0.05.

**Results:**

We observed 68 attempts by 34 subjects. Twenty-two (32.5%), 22 (32.5%), and 24 (35%) intubations were performed with the monitor positioned at the head, left, and right, respectively. The median times to recognition of hypoxia were 37 seconds [sec] (95% CI, 25–86 sec) for the head, 32 sec (95% CI, 18–50 sec) for the left, and 23 sec (95% CI, 19–34 sec) for the right groups, respectively. Cox regression demonstrated hazard ratios of 2.7 and 3.2 for the left and right groups when compared to the head group, and these findings were statistically significant (P = .04 and P = 0.03, respectively). We found no statistically significant associations between time to recognition of hypoxia and laryngoscopy modality or total number of previous intubations (P = .82 and .21, respectively). Failure rates across head, left, and right groups were similar, at 36%, 59%, and 50%, respectively (P = .30).

**Conclusion:**

Positioning of the patient monitor at the head of the bed results in delayed visualization of desaturation events during simulated direct and video-assisted laryngoscopy across different levels of experience. Ideally, the patient monitor should be positioned on the side of the bed and within the operator’s direct line of sight. Further research is warranted to assess how equipment setup may impact procedural performance, operator ergonomics, and patient safety.

## INTRODUCTION

Rapid sequence intubation (RSI) is one of the most important life-saving procedures performed by emergency physicians. In the emergency department (ED) setting RSI is typically performed in a stepwise, procedural manner with preoxygenation immediately preceding administration of medications to sedate and paralyze the patient. Following the administration of the induction agent and, more definitively, the paralytic, the patient becomes apneic requiring that vital signs, including oxygenation status, be monitored throughout the intubation attempt. Vital signs can readily be displayed on a patient monitor allowing them to be visualized and interpreted by the operator in real time.

Commonly, the patient monitor is placed and fixed at the head of the patient’s bed against the rear wall of the examination room. This optimizes the physical floor plan, facilitating access to the patient and ease of ingress and egress. Additionally, this setup allows the team leader who traditionally stands at the foot of the bed to assess vital signs during clinical encounters and resuscitation efforts. When a physician is required to intubate a patient independently, they are typically positioned at the head of the bed facing the patient and, thus, have their back to the patient monitor. While intubating in this position, visualization of the patient monitor becomes difficult, which may lead to missed or delayed recognition of abnormal vital signs, procedural inefficiencies, and operator discomfort.

Several key insights into the ergonomics of endotracheal intubation are provided in the literature; however, they are limited to optimizing bed height, operator posture, and patient position. A randomized controlled trial demonstrated that xiphoid-level positioning of the patient’s bed significantly reduced peak and impulse laryngoscopy forces while also improving laryngeal view and operator comfort compared to when the bed is positioned at the level of the anterior superior iliac spine.^1^ A pilot study investigating the impact of operating table heights on the quality of laryngeal view and operator discomfort during endotracheal intubation demonstrated that higher operating table positions (at the xiphoid process and nipple level of the operator) provided superior laryngeal view grades and required smaller ranges of neck and lower back flexion corresponding to less subject-reported discomfort compared to table heights at the level of the umbilicus.^2^

In terms of patient positioning, evidence remains conflicting. The 2023 Society of Critical Care Medicine guidelines suggest that inclining the patient’s head and trunk to the semi-Fowler position (20–25 degrees) may increase first-pass success by improving the glottic view, reducing aspiration risk, and increasing the time to desaturation as opposed to the sniffing and neutral positions.^3^ However, in a multicenter, randomized trial involving pulmonary and critical care fellows performing endotracheal intubation in the intensive care unit setting, the semi-Fowler position did not improve oxygenation and worsened the Cormack-Lehane grade of glottic view, resulting in lower first-pass success and a higher total number of intubation attempts compared to the sniffing position.^4^ In obese patients specifically, back-up head-elevated positioning has been suggested to reduce the number of intubation-related complications^5^; however, the nonsupine positioning of patients undergoing endotracheal intubation in the ED was associated with higher odds of any adverse event compared to the supine position.^6^

**Population Health Research Capsule**

What do we already know about this issue?

*Patient monitors are typically fixed at the head of the bed during intubation, requiring operators to turn away from the patient to view vital signs*.

What was the research question?

*Does position of the patient monitor affect the time to recognition of hypoxia during endotracheal intubation?*

What was the major finding of the study?

*Side-of-bed monitors improved hypoxia recognition vs head-of-bed location (hazard ratio 2.7–3.2)*.

How does this improve population health?

*Optimizing equipment positioning may reduce delayed recognition of hypoxia, potentially decreasing peri-intubation complications and improving patient safety*.

While the importance of strategic setup during emergency procedures is recognized, research and standardized guidelines regarding the optimal positioning of equipment and devices during RSI remain limited. The National Association of EMS Physicians recommends continuous physiological monitoring during airway management to guide the timing of, limit the duration of, and inform decision-making during the performance of advanced airway insertion techniques. Guidelines include continuous monitoring of the patient’s vital signs including pulse oximetry, heart rate, and blood pressure, but they do not provide specific details regarding how these measurements are to be recorded and displayed to allow for physician interpretation.^7^ The 2012 Eastern Association for the Surgery of Trauma practice management guidelines recommend the monitoring of pulse oximetry and end-tidal carbon dioxide monitoring as Level 1 enhancements for safe and effective intubation in trauma patients, but they offer no recommendations regarding equipment setup or operator positioning to optimize procedural ergonomics.^8^

A review of several published airway checklists for RSI in the ED revealed that while preparation- phase items typically cover patient assessment, equipment verification, personnel assignment, and backup planning, few mention equipment postioning.^9^–^12^ In fact, the only airway checklist we reviewed that mentioned the patient monitor as part of its pre-arrival checklist delineated “monitors and video laryngoscope screen positioned appropriately”; however, a description of what constituted an appropriate position was omitted.^12^ Our primary aim in this study was to investigate optimal patient monitor positioning during endotracheal intubation as determined by the operator’s ability to visualize a desaturation event. A secondary aim was to compare operator self-reported use of the patient monitor across various positions. We hypothesized that positioning the patient monitor within the direct line of sight of the operator throughout the intubation attempt would be associated with a shorter time to recognition of hypoxia while alleviating some of the technical challenges associated with the procedure.

## METHODS

### Study Design and Setting

We performed a multicenter, randomized crossover trial of emergency medicine (EM) resident trainees. The research intervention was implemented and subjects were observed in simulation centers located on the academic campuses of the respective recruitment sites. Each simulation center occupies over 4,000 square feet of physical space and contains state-of-the-art equipment, clinical simulation bays, and audiovisual capture learning management systems operated and maintained by full-time staff with advanced training in medical simulation. The room setup and equipment positions were standardized across sites prior to subject enrollment.

### Selection of Participants

Subjects were selected from the pool of resident trainees across two Accreditation Council for Graduate Medical Education-accredited EM residency programs. Site A hosts a 3-year residency program in EM with 48 trainees while Site B hosts a 4-year residency program in EM with 76 trainees. Both residency programs are affiliated with large, urban, academic healthcare systems with Level I trauma center certification in the northeast United States, and each healthcare system reports an annual census of approximately 110,000 adult ED patient visits. Subject participation was voluntary. An email containing information regarding the scope of the study, subject eligibility, and recruitment information was disseminated through established department listservs. The principal investigator’s contact information was made available prior to the consent process and after study completion. Subjects had an opportunity to withdraw from the study or withdraw data prior to analysis at any time and without consequence. The study was reviewed and approved by the affiliate institutional review boards (132564-39).

### Intervention

Each subject was asked to perform endotracheal intubation a total of two times on a Deluxe Difficult Airway Trainer (Laerdal Medical, Stavanger, Norway) in a simulated environment. Subjects performed endotracheal intubation using direct laryngoscopy (DL) once and video-assisted laryngoscopy (VL) once using standard geometry laryngoscope blades. The sequence of laryngoscopy modality (DL/VL or VL/DL) and the patient monitor position (head, left, or right of the patient bed) were randomized prior to each attempt. Vital signs were programmed using Laerdal Learning Application (LLEAP) simulation software and displayed on a 24-inch portable monitor. To standardize the height and position of the patient monitor screen between attempts and across sites, it was placed on a rolling cart 1.5 meters above the ground. The cart’s wheel positions were demarcated with tape on the floor. For all VL attempts, a GlideScope Core System (Verathon Inc., Bothell, WA) was mounted on a mobile stand and placed at the right of the patient bed.

For DL attempts, the GlideScope Core System was removed from the simulated space (Appendix, Supplemental Material 1). The tongues of the difficult airway trainers were fully insufflated to ensure maximum difficulty when attempting visualization of the glottic opening and mechanical passage of the endotracheal tube to prolong the duration of the attempt as much as possible. Prior to each attempt, a script describing the clinical scenario and requested action was read to the subject by a member of the research team (Appendix, Supplemental Material 2). Subjects were asked to intubate the patient as quickly as possible while addressing and responding to the patient’s condition as they would in a real clinical scenario.

Simulated patient vital signs including systolic and diastolic blood pressures, heart rate, respiratory rate, temperature, and peripheral capillary oxygen saturation (SpO_2_) were displayed on the patient monitor. The simulated patient’s SpO_2_ was programmed to begin at 100% and decrease at a rate of 1% per second 10 seconds after the start of the subject’s attempt. Pulse oximetry pitch tone was muted. The scenario would end if one of three conditions was satisfied: 1) the subject successfully intubated the manikin; 2) the subject aborted the intubation attempt and began to reoxygenate the patient; or 3) the subject surrendered. All attempts were recorded using audiovisual capturing equipment, and these recordings were saved on local password-protected servers.

Research personnel (EB, RP) independently reviewed the recordings for all subjects, and final measurements were averaged. If measurements had differed by > 5%, recordings for that subject would have been reviewed and measurements adjudicated by a third member of the research team (JB). None of the measurements differed by > 5%, and there was no need for a formal adjudication process. Immediately after each attempt, the subject was asked to complete a survey consisting of five statements regarding their experience and use of the patient monitor (Appendix, Supplemental Material 3). Subjects were asked to self-report their agreement with the statements on a 5-point Likert scale ranging from 1 (strongly disagree) to 5 (strongly agree).

Statements featured on the survey were as follows: 1) “I utilized the patient monitor effectively during the attempt”; 2) “I found it easy to visualize the patient monitor during the attempt”; 3) “I looked at the patient monitor at least once during the attempt”; 4) “I am confident that a desaturation event did not occur during the attempt”; and 5) “I am confident that a desaturation event did occur during the attempt.” Subjects were also asked to report their postgraduate year, estimated total number of previous intubations performed (< 50, 50–100, > 100), and handedness (right, left, ambidextrous).

### Measurements and Outcomes

Patient monitor position (head, left, right) and laryngoscopy modality (DL, VL) were recorded as categorical variables. We defined the primary outcome—time to recognition of hypoxia—by the observed period during which the simulated patient’s SpO_2_ displayed on the monitor was < 90% and recorded as a continuous variable in seconds (sec). Scenario outcomes including failure, reoxygenation attempt, and surrender were categorized as binary variables. Previous studies have described that performing 50 intubations is enough to become proficient, while performing 100 intubations is required to reach mastery level.^13^–^14^ For that reason, we recorded the estimated total number of previous intubations performed as a categorical variable (< 50, 50–100, > 100), which we selected as a proxy for operator level of experience. We believe that the total number of previous intubations performed is a more valid measure of procedural proficiency than postgraduate year, as exposure to and familiarity with procedures during residency training are experience-dependent and may not strongly correlate with year of training.

### Sample Size Estimation

Previous reports in the literature describe a median laryngoscopy duration of 35 sec, and attempts in which VL was used were 6 sec longer.^15^ There are no pre-existing data describing the association between patient monitor position and duration or likelihood of a desaturation event when performing endotracheal intubation. Through expert consensus, we hypothesized that the desaturation duration would be shorter when the patient monitor was positioned to the left or right as compared to the head of the patient bed. Using a two-tailed test with a desired power of 80% and an alpha equal to 0.05, a total of three subjects would be required if the mean desaturation duration for the left and right groups were 25 sec, while 11 subjects would be required if the mean desaturation duration were 30 sec. Because we had planned to include patient monitor position, estimated total number of previous intubations performed, and laryngoscopy modality in the final regression model, we targeted a sample size of 50.^16^

### Statistical Analysis

We summarized subject characteristics and procedural performance measures using descriptive statistics. Shapiro-Wilk tests were applied to determine whether the distribution of data was normal. To assess the primary aim, Kaplan-Meier curves illustrating the time to visualization of a desaturation event were rendered, and we analyzed a Cox regression, adjusting for laryngoscopy modality and estimated total number of previous intubations performed. Given that subjects performed the intervention twice, we used a shared frailty model to account for within-cluster dependence. Additionally, we performed a multivariable linear regression analysis that included laryngoscopy modality and estimated total number of previous intubations performed as covariables.

To assess differences in the number of observed failure events, we used chi-squared or Fisher exact tests. For the secondary aim comparing differences in subject survey responses, Cochran-Mantel-Haenszel tests for trend were performed for each question to test the null hypothesis that the proportions of the dependent variable were the same among the ordered exposure categories. Additionally, we used analysis of variance (ANOVA) testing to compare the mean survey response score across groups. We applied Tukey’s multiple comparison tests with Bonferroni correction for results statistically significant at the global level. The threshold for statistical significance was prespecified as a two-sided alpha level of 0.05, and all analysis was performed using SAS Studio v9.4 (SAS Institute Inc, Cary, NC).

## RESULTS

### Characteristics of Study Subjects

We observed 68 intubation attempts and included them in the analysis. Subject characteristics and procedural performance measures for the overall sample are summarized in Table 1. The overall failure rate was 48.5%. The median desaturation duration for the overall sample was 28.5 sec (95% CI, 22–36).

### Main Results

Twenty-two (32.5%), 22 (32.5%), and 24 (35%) intubations were performed with the patient monitor at the head, left, and right positions, respectively. Subject characteristics and procedural performance measures across groups are summarized in Table 2.

**Figure 1:**
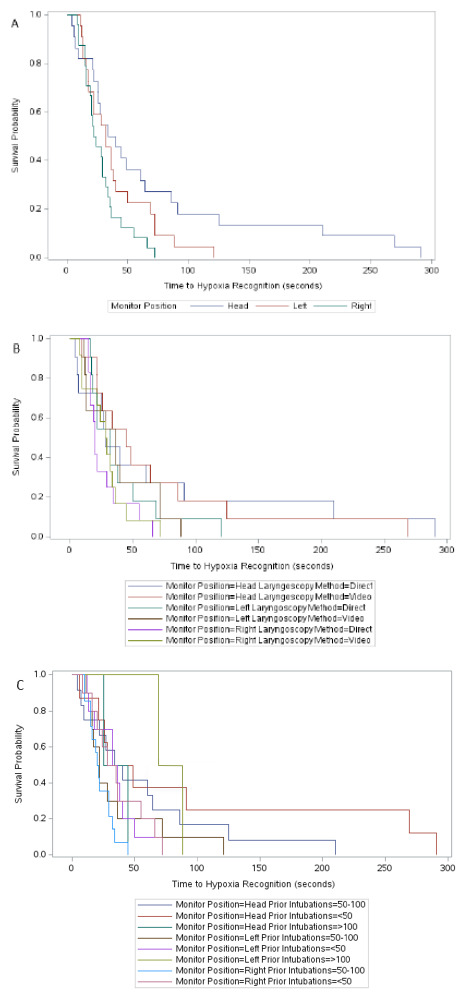
Kaplan-Meier curves illustrating the time to operator visualization of a desaturation event in a randomized controlled crossover trial (A) across groups for the overall sample, (B) across groups stratified by laryngoscopy modality, and (C) across groups stratified by estimated total number of previous intubations performed.

Kaplan-Meier curves illustrating the time to operator visualization of a desaturation event across groups for the overall sample and stratified by laryngoscopy modality and total number of previous intubations performed are provided in Figure 1.

Cox regression demonstrated hazard ratios of 2.7 and 3.2 for the left and right groups when compared to the head group, and these findings were statistically significant (*P* = .04 and *P* = .03, respectively). No statistically significant associations between time to hypoxia recognition and laryngoscopy modality or estimated total number of previous intubations performed were found (*P* = .82 and *P* = .21, respectively). The failure rates across head, left, and right groups were 36%, 59%, and 50%, respectively; these differences did not meet statistical significance (*P* = .30).

A summary of subject responses to the post-intervention survey questions is provided in Table 3. Cochran-Mantel-Haenszel tests for trend were statistically significant for questions 1 (*P* < .01), 2 (*P* <. .001), 3 (*P* <. .001), and 5 (*P* <.01). Global ANOVA demonstrated statistically significant differences in responses to questions 1, 2, 3, and 5. Pairwise comparisons demonstrated less effective utilization (H-L: −1.8, 95% CI, −2.6 to −0.9, *P* <.01, H-R: −1.2, 95% CI, −2 to −0.3, *P* <. .01); reduced ease (H-L: −2.8, 95% CI, −3.2 to −1.7, *P* < .01, H-R: −2.4, 95% CI: −3.2 to −1.7, *P* < .01); decreased overall utilization (H-L: −2.1, 95% CI, −3.1 to −1.1, *P* <0.01, H-R: −2.0, 95% CI, −3 to −1.1, *P* < .01) and reduced confidence in recognition of a desaturation event (H-L: −1.1, 95% CI, −2 to −0.2, *P* < .01, H-R: −1, 95% CI, −1.8 to −0.1, *P* < .01) when the monitor was positioned at the head as compared to left and right of the patient bed.

## DISCUSSION

Endotracheal intubation is a uniquely challenging procedure to perform, especially when faced with the difficult airway; however, it can be reproduced with high fidelity in the simulated environment.^17^–^18^ Almost all existing simulation studies surrounding intubation focus on the technical procedure itself and vary only slightly regarding technique, equipment used, clinical setting, and subject scope and level of training. While fiberoptic devices and VL have replaced DL as the preferred technique for first attempt in the ED setting in recent years,^19^–^20^ far less attention has been directed toward equipment positioning and its potential impact on operator ergonomics and situational awareness. In this study, we found that patient monitor position impacted the time to operator recognition of hypoxia, with head-of-bed positioning associated with significantly longer times compared with side-of-bed placement. Mean desaturation events approached 70 sec with head placement, compared to 39 sec for left placement and 28 sec for right placement. Although this study was conducted in a simulated environment, similar delays in real-world clinical scenarios could increase the risk of peri-intubation complications, particularly in critically ill patients with limited physiological reserve.

When the patient monitor was located at the head of the bed, operators were required to rotate their necks and torsos to view the display. This additional effort likely reduced the frequency and efficiency of monitoring vital signs during the intubation attempt. Additionally, the patient monitor is not in the periphery of the operator’s visual field, making it less likely to draw attention when abnormal measurements are displayed. Importantly, these differences persisted regardless of laryngoscopy modality and operator level of experience, suggesting that even skilled EM residents remain subject to the ergonomic limitations of suboptimal patient monitor placement. We expected shorter times to hypoxia recognition for subjects reporting the highest estimated total number of previous intubations performed as clinical experience has been shown to correlate with situational awareness even in simulated environments.

Given that DL is more physically challenging and technically difficult than VL, as it requires the operator to bend forward and focus their attention on the patient’s airway instead of objects in the environment, we were surprised to find no statistically significant association between time to hypoxia recognition and laryngoscopy modality. Not surprisingly, times were shortest during attempts when the patient monitor was positioned to the right of the bed, as the screens displaying pertinent clinical information were consolidated in a smaller area of the operator’s visual field, thus requiring minimal body movement and gaze switching.

Ergonomics research in surgical and occupational domains provides insight into why patient monitor positioning may influence performance during advanced airway management. In minimally invasive surgery, for example, misalignment between the operator’s visual display and task axis has been shown to impair technical efficiency, increase error rates, and heighten musculoskeletal strain.^21^ Displays placed outside the natural line of sight require awkward head or torso movements, which add both physical and cognitive constraints. Similarly, ergonomic studies of visual display terminals have demonstrated that neutral or slightly downward gaze angles (approximately 0–15 degrees below horizontal) reduce cervical strain and improve efficiency, whereas lateral or upward gaze increases musculoskeletal load and slows recognition of visual information.^22^

Regarding the subject experience, survey responses illustrated that head-of-bed monitor positioning significantly limits its effective use, which translated into uncertainty surrounding the patient’s clinical status. Repositioning the patient monitor to the left or right of the patient bed may facilitate its ease of use, thus increasing the frequency of vital sign reassessment, improving the likelihood of recognizing a desaturation event in a timely fashion, and increasing the physician’s confidence surrounding clinical decisions. In high-stress critical airway management scenarios, where operators must perform complex technical maneuvers and monitor and interpret patient physiology simultaneously, such disadvantages may exacerbate cognitive load and delay recognition of critical events. We believe this phenomenon would be most pronounced in clinical scenarios that involve a single physician responsible for both resuscitating and performing procedures independently and without the support of an interdisciplinary ancillary team.

From a practical perspective, our results highlight the importance of careful consideration of equipment placement and installation when configuring ED patient-examination rooms and resuscitation bays. Adjustable ceiling-mounted or articulated monitors, which have been shown to better accommodate ergonomic gaze angles across different postures and body movements, may be preferred.^23^ When such modifications are unavailable or impractical due to physical or financial constraints, positioning patient monitors at the side of the bed rather than at the head may serve as a simple solution. Incorporating ergonomic guidelines into standardized airway setup protocols and checklists may improve both procedural performance, operator ergonomics, and patient outcomes.

Lastly, our findings raise important considerations for airway management training. Teaching resident trainees to deliberately optimize patient monitor positioning may instill safer habits that persist into clinical practice. Furthermore, future studies should explore how patient monitor position may impact other operator factors, such as stress, fatigue, efficiency, satisfaction, and communication during advanced airway management. Integrating technologies such as motion capture, electromyography, or eye tracking could provide objective data linking head movement, posture, and gaze behavior to recognition delays and provide feedback regarding optimal operator biomechanics.

## LIMITATIONS

There are several limitation to this study. Subjects consisted of only resident trainees from two EM training programs affiliated with large, academic healthcare systems with Level 1 trauma centers in the northeast U.S. This may limit generalizability across ED settings, geographic regions, scope of training, operator experience levels, and clinical specialties. Further, the research intervention was conducted in a simulated environment, which may not fully replicate the complexities and nuances of real-life clinical scenarios. The simulated desaturation rate of 1% per second beginning 10 seconds following the initiation of the intubation attempt and the maximally insufflated tongue of the difficult airway trainer may not match all clinical scenarios, which also affect the generalizability and transferability of results. The short period of time preceding desaturation facilitated data collection and ensured that simulation center resources were used efficiently. The maximally insufflated tongue ensured that the intubation attempt would be difficult, so that the scenarios would not end prior to the programmed desaturation event occurring.

The simulated intervention was designed to ensure standardization between subjects and across sites. The study incorporated state-of-the-art equipment stored and maintained in simulation centers with full-time staff trained and certified in conducting medical simulation. The pulse oximetry pitch tone was muted during subject attempts, and only statements regarding visualization of the patient monitor in the absence of auditory feedback could be made.

## CONCLUSION

In this study we sought to enhance our understanding of how patient monitor positioning influences emergency physician procedural performance and experience during endotracheal intubation. The positioning of the patient monitor during endotracheal intubation significantly impacts the ease of its visualization and likelihood of its use in a simulated single-clinician scenario with pulse oximeter pitch tone muted. Fixation of the patient monitor at the head of the patient bed behind the turned back of the operator can impede visualization and timely recognition of desaturation events during both direct and video laryngoscopy and across varying levelss of EM resident skill. Further research into how positioning of the patient monitor during endotracheal intubation may optimize procedural performance, operator ergonomics, and patient outcomes in real-world clinical environments is warranted.

## Supplementary Information







## Figures and Tables

**Table 1:** Characteristics of study subjects and procedural performance measures for the overall sample in a randomized controlled crossover study investigating the effects of monitor positioning on time to operator visualization of a desaturation event.

Characteristic	Overall (N = 68)
Level of training (n, %)	
PGY-1	20 (29.4)
PGY-2	18 (26.5)
PGY-3	20 (29.4)
PGY-4	10 (14.7)
Total number of previous intubations performed (n, %)	
< 50	28 (41.2)
50–100	36 (52.9)
> 100	4 (5.9)
Handedness (n, %)	
Right	58 (85.3)
Left	8 (11.8)
Ambidextrous	2 (2.9)
Laryngoscopy Modality (n, %)	
Direct	34 (50)
Video-assisted	34 (50)
Mean desaturation duration, seconds (95% CI)	45.2 (32.4–58.1)
Median desaturation duration, seconds (95% CI)	28.5 (22–36)
Observed failure (n, %)	33 (48.5)
Observed reoxygenation (n, %)	35 (51.5)
Observed surrender (n, %)	0 (0)

*PGY*, postgraduate year.

**Table 2:** Characteristics of study subjects and procedural performance measures across groups in a randomized controlled crossover study investigating the effects of monitor positioning on operator ability to visualize a desaturation event.

Characteristic	Head (n = 22)	Left (n = 22)	Right (n = 24)
Level of training (n, %)			
PGY-1	4 (20)	8 (40)	8 (40)
PGY-2	6 (33.3)	2 (11.1)	10 (55.6)
PGY-3	8 (40)	6 (30)	6 (30)
PGY-4	4 (40)	6 (60)	0 (0)
Total number of previous intubations performed (n, %)			
< 50	8 (28)	10 (36)	10 (36)
50–100	12 (33.3)	10 (27.8)	14 (38.9)
> 100	2 (50)	2 (50)	0 (0)
Handedness (n, %)			
Right	20 (34.5)	20 (34.5)	18 (31)
Left	2 (25)	0 (0)	6 (75)
Ambidextrous	0 (0)	2 (100)	0 (0)
Laryngoscopy modality (n, %)			
Direct	11 (32)	11 (32)	12 (36)
Video-assisted	11 (32)	11 (32)	12 (36)
Mean desaturation duration, seconds (SD)	70 (82.7)	39 (28.8)	28 (16.9)
Median desaturation duration, seconds (IQR)	37 (22–86)	32 (17–50)	23 (16–34.5)
Observed failure (n, %)	14 (40)	9 (25.7)	12 (34.3)
Observed reoxygenation (n, %)	8 (24)	13 (40)	12 (36)
Observed surrender (n, %)	0 (0)	0 (0)	0 (0)

*IQR*, interquartile range*; PGY*, postgraduate year; *SD*, standard deviation.
