# Supplementary material for: Patient Monitor Position and Operator Ability to Visualize a Desaturation Event During Intubation
Source: West J Emerg Med. 2026 Jun 16;27(4):956–63. doi: 10.5811/westjem.52933 (PMC13436668; doi:10.5811/westjem.52933)
Supplement: Supplementary file 1 [file wjem-27-956-s001.docx]

**Supplemental Material 1.** Diagram illustrating standardized room and equipment setup.

Abbreviations: subject (S), patient bed (P), patient monitor position – head of bed (A), patient monitor position – left of bed (B), patient monitor position – right of bed (C), GlideScope® Core System position (G)
