# Supplementary material for: Patient Monitor Position and Operator Ability to Visualize a Desaturation Event During Intubation
Source: West J Emerg Med. 2026 Jun 16;27(4):956–63. doi: 10.5811/westjem.52933 (PMC13436668; doi:10.5811/westjem.52933)
Supplement: Supplementary file 2 [file wjem-27-956-s002.docx]

**Supplemental Material 2.** Introductory script describing the clinical scenario and requested action read by research personnel prior to each subject attempt.

To be read by research personnel prior to each attempt:

**During this scenario, you are asked to perform an endotracheal intubation on the patient before you with the equipment provided. Please attempt to complete the procedure as quickly as possible and to the best of your ability, acknowledging and responding t the patient’s condition as you would in a real clinical practice setting. You may assume that the patient has been optimally preoxygenation, appropriately positioned, and adequately sedated and paralyzed prior to start. At this time, please familiarize yourself with the equipment and room setup, but please maintain the items in their current position.**
