# Supplementary material for: Patient Monitor Position and Operator Ability to Visualize a Desaturation Event During Intubation
Source: West J Emerg Med. 2026 Jun 16;27(4):956–63. doi: 10.5811/westjem.52933 (PMC13436668; doi:10.5811/westjem.52933)
Supplement: Supplementary file 3 [file wjem-27-956-s003.docx]

**Supplemental Material 3.** Post-intervention survey consisting of 5 statements regarding the subject experience and patient monitor utilization during the intubation attempt. Subjects were asked to self-report their agreement with the statements on a 5-point Likert scale ranging from 1 (strongly disagree) to 5 (strongly agree).
